# Letter to the editor on “Geotemporospatial and causal inferential epidemiological overview and survey of USA cannabis, cannabidiol and cannabinoid genotoxicity expressed in cancer incidence 2003–2017, parts 1–3”

**DOI:** 10.1186/s13690-022-00932-0

**Published:** 2022-07-19

**Authors:** Frank Yizhao Chen, Michael Barnes, Evan Cole Lewis

**Affiliations:** 1 Jamaican Medical Cannabis Corporation, Toronto, Canada; 2 Medical Cannabis Clinicians Society, London, UK; 3 Neurology Centre of Toronto by Numinus, 419 Eglinton Avenue West, Suite 100, Toronto, ON M5N1A8 Canada

**Keywords:** Cannabis, Cannabinoids, Cancer, Causal role, Confounders, Ecological study, Observational study, Epidemiological methodology, Methodological appraisal

## Abstract

We would like to thank authors Reece and Hulse (2022) for their three-part article titled “Geotemporospatial and causal inferential epidemiological overview and survey of USA cannabis, cannabidiol and cannabinoid genotoxicity expressed in cancer incidence 2003-2017”, in which the authors infer that cannabis use has a causal role in the development of various cancer types. While the authors use reputable datasets and a well-established epidemiological methodology, the authors’ conclusion of a causal association is limited due to biases inherent in ecological epidemiological studies. Though the researchers attempt to overcome these biases through validation and statistical manipulations, their approaches are insufficient to create conditions suitable for causal inferencing upon examination. There are also concerns in the practical and conceptual application of the studies’ dataset that further question the validity of the authors’ inferences. Further research exploring the potential benefits and harm of cannabinoids in the context of cancer must be performed before a distinct relationship can be defined.

## Main text

A three-part study by Reece and Hulse [1] that associated cannabis with a causal role to cancer has recently been published. Though it uses reputable American datasets and multiple analysis models that meet foundational epidemiological standards, the claim of causal inferences linking cannabis to cancer is unsubstantiated. The study design limits causal inferences, and the attempts to overcome these limitations are insufficient. There are also issues in the dataset that further question the findings.

The employed epidemiological study’s design is inherently limited in inferring causation. The primary data and findings are in the form of state-level trends in cancer rates and drug exposure, yet the researchers infer causal dose-response physiological cannabinoid mechanisms and individual risk of developing of cancer. These interpretations are clear examples of ecological fallacy – a bias that falsely assumes population-level results imply individual-level effects – and should be taken with caution.

The authors address the ecological fallacy through validation and statistics but fail to create conditions for causal inference. For instance, the authors validate their techniques’ causal interpretations by replicating well-known relationships between tobacco and various cancer types. However, this is threatened by a failure to replicate other well-established drug-cancer associations, like those between alcohol and liver and oropharyngeal cancers. The validation is also weakened by inattention to research showing that cannabinoids may have antitumor effects in various cancer types [2]. The authors’ reliance on statistical manipulations – E-values and inverse probability weighting (IPW) – also falls short in allowing causal interpretations. The controlling of four confounders (age, state-level household incomes, ethnicities, population) in a complex relationship involving multiple factors (e.g. migration of individuals between states; multi-drug usage, etc.) affects the reliability of E-values and IPW. E-values should be synergistically used with efforts to control for as many confounders as possible [3]. IPW can only account for measured confounders [4]. It is also concerning, out of 28 cancer types explored, the authors applied their strongest statistical method to only two – prostate and ovarian. Plus, like all statistical tests, E-values and IPW should be used as additional assessments of evidence [3, 4], and cannot confidently “imply causality” [1].

There are concerns that the datasets Reece and Hulse used are inappropriately applied. The generalization of cannabis cannabinoid content data across US states is concerning, because cannabis composition may vary [5]. This cannabinoid dataset is also inappropriately generalized by including a significant presence of regulated US cannabis, given that the cannabinoid content was derived from illegal Drug Enforcement Agency-seized cannabis (DSC). DSC is unregulated in cannabinoid composition and may contain other harmful constituents. Its use for medical or recreational purposes is also uncertain, potentially lending itself to a disproportionate amount being inhaled via smoking rather than consumed in another less harmful manner. Compare this to regulated cannabis by which its composition is carefully controlled and is consumed through a variety of routes of administration, especially since its use is distributed across medical and recreational indications.

The researchers’ contemporaneous analysis of cancer rates and drug exposures also presents a conceptual issue, as it falsely assumes cancer diagnoses occurs within a year of drug exposure. Even for the cancer types the authors correlated with cannabis use – prostate and ovarian – that were adjusted for temporal lag, their 10-year models may be too narrow. Age groups between 18 and 34 account for a significant proportion of cannabis use [6], while ovarian and prostate cancer have median diagnoses at 50–79 [7] and 67 years [8], respectively.

While Reece and Hulse use well-established study design and methodology, the approach is misdirected in claiming a causal role for cannabis use in the development of cancer – the conclusion must be called into question. A failure to overcome inherent study design characteristics and concerns about the application of the dataset to the question prevent the establishment of a causal relationship from being inferenced. Further in-depth research on the relationship between cannabinoids and cancer is required before a harmful causal role is established or inferred.

## Data Availability

N/A.

## Abbreviations

DSC: Drug Enforcement Agency-Seized Cannabis
IPW: Inverse Probability Weighting
USA: United States of America
